# Realizing nearly-free-electron like conduction band in a molecular film through mediating intermolecular van der Waals interactions

**DOI:** 10.1038/s41467-019-11300-y

**Published:** 2019-07-29

**Authors:** Xingxia Cui, Ding Han, Hongli Guo, Linwei Zhou, Jingsi Qiao, Qing Liu, Zhihao Cui, Yafei Li, Chungwei Lin, Limin Cao, Wei Ji, Hrvoje Petek, Min Feng

**Affiliations:** 1 School of Physics and Technology and Key Laboratory of Artificial Micro- and Nano-Structures of Ministry of Education, Wuhan University, Wuhan, 430072 China; 2 Beijing Key Laboratory of Optoelectronic Functional Materials & Micro-Nano Devices, Department of Physics, Renmin University of China, Beijing, 100872 China; 3 Mitsubishi Electric Research Laboratories, 201 Broadway, Cambridge, MA 02139 USA; 4 Department of Physics and Astronomy and Pittsburgh Quantum Institute, University of Pittsburgh, Pittsburgh, PA 15260 USA; 5 Institute for Advanced Studies, Wuhan University, Wuhan, 430072 China

**Keywords:** Carbon nanotubes and fullerenes, Electronic properties and materials, Molecular self-assembly, Surfaces, interfaces and thin films

## Abstract

Collective molecular physical properties can be enhanced from their intrinsic characteristics by templating at material interfaces. Here we report how a black phosphorous (BP) substrate concatenates a nearly-free-electron (NFE) like conduction band of a C_60_ monolayer. Scanning tunneling microscopy reveals the C_60_ lowest unoccupied molecular orbital (LUMO) band is strongly delocalized in two-dimensions, which is unprecedented for a molecular semiconductor. Experiment and theory show van der Waals forces between C_60_ and BP reduce the inter-C_60_ distance and cause mutual orientation, thereby optimizing the π-π wave function overlap and forming the NFE-like band. Electronic structure and carrier mobility calculations predict that the NFE band of C_60_ acquires an effective mass of 0.53–0.70 *m*_e_ (*m*_e_ is the mass of free electrons), and has carrier mobility of ~200 to 440 cm^2^V^−1^s^−1^. The substrate-mediated intermolecular van der Waals interactions provide a route to enhance charge delocalization in fullerenes and other organic semiconductors.

## Abstract (web summary)

Fullerenes are promising organic semiconducting materials for electronic applications, but of low conductivity due to limited intermolecular hybridization. Cui et al. show a black phosphorous substrate organizes C_60_ monolayers to form delocalized semiconducting band with tens times enhanced conductivity.

## Introduction

The degree of electron delocalization in organic semiconductors is critical for their adoption in electronic and optoelectronic applications^1–3^. Electron transport, however, is usually facile through chemical bonds in conjugated organic materials, but is rarely optimal when the noncovalent intermolecular van der Waals (vdW) forces define the self-assembly and consequently, the intermolecular electronic coupling^4^. C_60_ molecules, which have uncommonly large electron affinity and suitable electronic band gap, have been extensively investigated as zero- to three-dimensional (0-3D) organic semiconductors^5–9^. As a typical vdW material, C_60_ molecules form solids through a balance of the Pauli type, electrostatic repulsive force and the attractive vdW force^10^, which, however, does not enhance intermolecular electronic hybridizations. C_60_ solids are characterized by  flat electronic bands with inconsequential dispersions and hopping transport. Functionalization of C_60_ by synthesizing fullerene derivatives has been investigated as a means to improve its electron transport, but the improvement was limited as the dispersions of the electronic bands were not effectively modified^11–14^. A radical improvement of electron transport, however, might be achieved if the assembly of C_60_ molecules could increase the intermolecular electronic hybridizations.

Even though the vdW forces are weak compared to other bonding mechanisms, it can have a profound impact on the electronic properties. For example, a recent study has demonstrated that by controlling the vdW interactions in a bilayer graphene, it is possible to transform a nominally semiconducting material into a superconductor^15^. Our method to mediate the vdW interactions between C_60_ molecules is through a substrate control of intermolecular interactions. It has been demonstrated that epitaxial growth of CO molecules on metals can impose an in-plane molecular compression that affects the intermolecular electronic interactions^16,17^. On metal surfaces, however, the high electron affinity of C_60_ enables it to naturally draw electrons from its substrates and thereby to become metallic^18,19^. Thus, to retain and enhance its semiconducting properties, it is far more desirable to impose intermolecular interactions on C_60_ molecules with an electronically inert substrate. From this point of view, black phosphorous (BP)^20,21^, composed of atomic sheets that are held by vdW forces with alternating projecting “ridge” and subsiding “notch” atoms in a single layer, provides a unique adsorption platform for C_60_ molecules with yet unknown consequences.

Here we show by scanning tunneling microscopy (STM) and theory that the BP substrate organizes C_60_ molecules into a compressed monolayer and imposes a favorable orientation that optimizes the intermolecular π-π couplings, resulting in a nearly-free-electron (NFE) like lowest unoccupied molecular orbital (LUMO) band in C_60_ monolayers. Such NFE-like LUMO band would dominate charge transport in C_60_ assemblies. An NFE band in C_60_ nanostructures has been attested through discovery of the superatom molecular orbitals (SAMOs)^22^, and their strong hybridization. But SAMOs and their NFE bands are too high in energy to participate in charge transport unless C_60_ molecules are endohedrally doped with metallic atoms^23^. NFE dispersion has been attributed to π orbitals of a molecular monolayer on metallic substrates, but its origin has later been reassigned to quantum confinement of a metal surface state, rather than the electronic hybridization among organic molecules^24–28^. The NFE conduction formed by π-π interactions is worthy of further exploration in C_60_ or other organic molecules on inert substrates, because it realizes a long sought mode for charge transport for high-performance organic electronics and optoelectronics^4^.

## Results

### Topography of C_60_ monolayer on BP

**Fig. 1:**
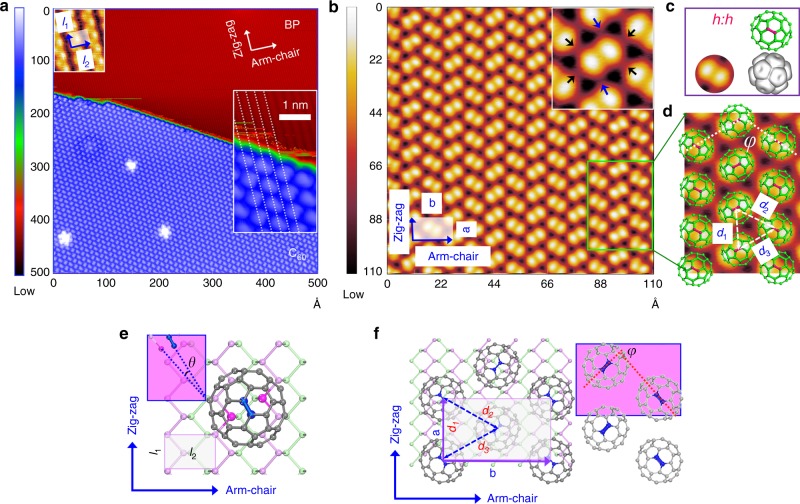
The adsorption structure and site of C_60_ on BP. **a** STM image (*I* = 30 pA, *V*_b_ = −1.55 V) of an ordered C_60_ monolayer island on BP. The upper left inset shows an atomic resolution STM image (*I* = 60 pA, *V*_b_ = −0.60 V) of the bare BP surface. Only the upper atoms of the topmost puckered BP layer are observed. The surface unit cell is marked with *l*_1_ and *l*_2_ representing the lattice constants along zig-zag and arm-chair directions, respectively. The lower right inset shows an enlargement at a termination of a C_60_ island: The dashed white lines crossing the center of close-packed C_60_ molecules extend to the location of “notches” of the BP surface. **b** A close-up STM image (*I* = 30 pA, *V*_b_ = 1.05 V) of C_60_ on BP that shows two-lobes of intramolecular contrast. The inset images show six locations of moderately enhanced contrast around each C_60_ molecule which can be categorized into two kinds by symmetry along the arm-chair direction (indicated by black arrows) and the zig-zag direction (indicated by blue arrows). **c** The model structure shows that C_60_ adsorbs on the BP surface with the *h*:*h* bond of C_60_ icosahedra pointing up. **d** Enlargement of the white rectangle in (**b**). C_60_ ball-and-stick model is superimposed over C_60_ images to highlight the experimental orientation of *h*:*h* bonds, which alternates with a relative angle of *φ* = 99.2º ± 0.4º. **e** A ball-and-stick model of the most stable adsorption structure of isolated C_60_ on BP obtained from DFT calculations. The rectangle represents the calculated unit cell of BP with *l*_1_ and *l*_2_ dimensions as in (**a**). The C and P atoms involved in the interaction are highlighted by deep blue and dark pink, respectively. The relative orientation of the of *h*:*h* bond to the BP substrate is indicated by *θ*. **f** A ball-and-stick model of the most stable adsorption structure of C_60_ lattice on BP obtained from DFT calculations. The unit cell of the C_60_ lattice is: *a* = 3 *l*_1_; *b* = 4 *l*_2_ and the calculated relative orientation of *h*:*h* bonds is *φ* = 97.3º. The calculated intermolecular distances are d_1_ = d_3_ = 9.9 Å and d_2_ = 10.07 Å. The STM images are obtained at 77 K

Figure 1a shows a topographic reconnaissance STM image of a C_60_ monolayer island on BP, where C_60_ molecules aggregate into a compact, highly ordered film. The top inset in Fig. 1a shows a topographic STM image, with atomic resolution, of the BP substrate where only the topmost ridge atoms are resolved while the lower notch atoms remain undetected within the dark waving lines. A rectangular surface unit cell is marked in the image with lattice constants *l*_1_ and *l*_2_ along the zig-zag and arm-chair directions, respectively. By analyzing the central position of C_60_ molecules at the island edge (bottom right inset in Fig. 1a), we establish that they assemble above notches of the BP layer (noted by white dashed lines), while the exact adsorption sites will be elaborated later. Figure 1b presents a close-up STM topographic image of unoccupied states (*V*_bias_ = +1.05 V) of the C_60_ monolayer, where each molecule appears as two bright lobes. As established in previous studies^18,29,30^, the characteristic two-lobe structure corresponds to the unoccupied π-orbitals of two pentagons that are joined by a C = C bond (see Fig. 1c). Here, we mark the C = C bond with “*h*:*h*” (The “*h*:*h”* designation represents C = C bonds shared by the two side-by-side hexagons)^31^. This two-lobe intramolecular feature establishes that C_60_ molecules sit on the surface in a highly ordered structure, where its two side-by-side hexagons and *h*:*h* joined pentagons point up (Fig. 1c).

In Fig. 1b, it is clear that along the zig-zag direction of the substrate, the *h*:*h* bonds are oriented in the same direction forming an equivalently oriented C_60_ chain over the BP notches. In the adjacent C_60_ chain, the *h*:*h* bonds are again oriented parallel to each other, but the *h*:*h*-*h*:*h* angle (indicated by *φ* in Fig. 1d) with respect to the original chain is 99.2 ± 0.4°, i.e., the *h*:*h* bonds orientation alternates between C_60_ molecules in the arm-chair direction. The alternation of the *h*:*h* bonds are highlighted in Fig. 1d, where ball-stick models of C_60_ are superimposed on the topography image with the *h*:*h* bonds marked in red. With this molecular arrangement, C_60_ molecules form a centered supercell, which contains two molecules of different *h*:*h* orientations with lattice constants *a* = 9.9 ± 0.2 Å (zig-zag) and *b* = 17.2 ± 0.2 Å (arm-chair), corresponding to three and four times the substrate lattice constants along the zig-zag and arm-chair directions, respectively. The obtained intermolecular distances *d*_1_ is nearly identical to *d*_2_ with values of 9.9 ± 0.2 Å, and *d*_3_ is of 10.0 ± 0.2 Å (Fig. 1d). These distance values imply a tighter packing of C_60_ molecules in the monolayer than that in molecular crystals where the vdW distance is 10.04 Å. The distance is also smaller than commonly found in C_60_ monolayer on most metal surfaces^29,32–36^. The STM image shown in the inset of Fig. 1b identifies six positions with weak, bright contrast around each C_60_ molecule, as marked by arrows; two arrows belong to interfaces between C_60_ molecules along the zig-zag direction (blue) and the rest are along the arm-chair direction (black). We will elucidate later how this interface contrast relates to strong intermolecular interactions unique to the C_60_ monolayer on BP surface.

Figure 1e presents the most stable adsorption structure of an isolated C_60_ molecule on a BP tri-layer based on DFT structural optimization. Consistent with the experiment, the bottom *h*:*h* bond (depicted in deep blue) lays over the BP notches such that each C atom interacts with two of the closest P atoms (dark pink). The relative orientation of the bottom *h*:*h* to the interacting P-P bond direction has an angle of *θ* = 11.0° (Fig. 1e). To get more insight into C_60_-BP interactions, we calculate the differential charge density [DCD, total – (BP + C_60_)] between C_60_ and BP. The largest DCD of 0.0029 eÅ^−3^ is about 0.001 of the largest pre-adsorption density of 2.1925 eÅ^−3^, implying there is negligible charge transfer between BP and C_60_ and the interaction mainly occurs through vdW forces. The adsorption gains −1.24 eV energy per C_60_ molecule and the C–P distances are 3.42~3.46 Å, consistent with the vdW force mediated adsorption energy of C_60_ on graphene^37^.

Figure 1f shows the most stable molecular configuration of the C_60_ monolayer predicted by DFT. The orientation of C_60_ molecules within the monolayer is similar to that of isolated C_60_ molecules on BP, with the bottom *h*:*h* bond almost parallel to the substrate P-P bond with an angle of 1.6° between them. These results indicate that condensing C_60_ into a monolayer does not strongly alter the local adsorption structure with respect to that of single C_60_ molecules, implying that C_60_-BP interactions dominate the monolayer formation. This assertion can also be quantified from an energetic perspective. The obtained absorption energy of −1.36 eV per C_60_ molecule in the monolayer on BP is only 0.12 eV larger than that for an isolated C_60_. The adsorption energy difference can be regarded as the energy gain due to inter-C_60_ interactions, which are much weaker than that for the BP–C_60_ interaction. Our calculations also reproduce the observed molecular arrangement with identical *h*:*h* bonds orientation along the zig-zag direction and alternating *h*:*h* bonds along the arm-chair direction. The theoretical lattice constants *a* and *b* of 9.90 Å (*d*_1_) and 17.20 Å and *φ* of 97.3° reaffirm the compressed C_60_ structure. We calculate that such in-plane lattice compression corresponds to an effective pressure of 1.8–2.2 GPa on a C_60_ monolayer by the BP–C_60_ interaction.

### Delocalized LUMO state of C_60_ on BP surface

**Fig. 2:**
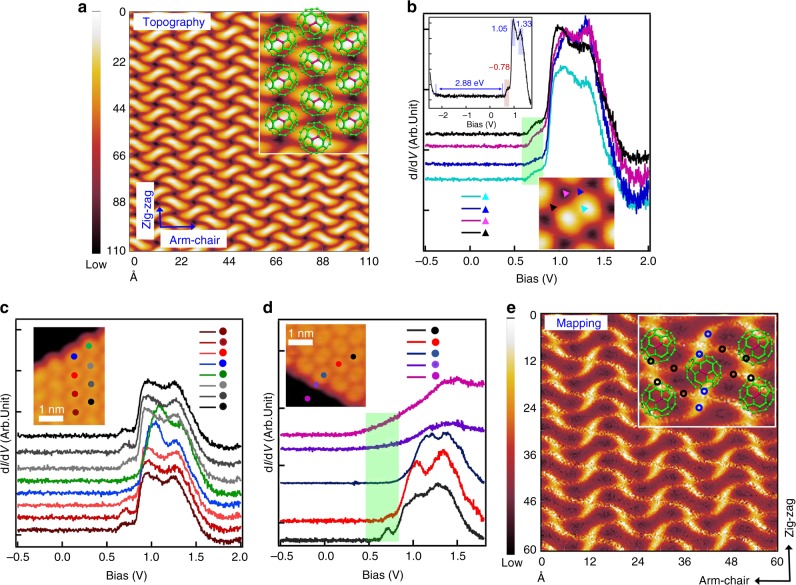
Highly delocalized LUMO state of C_60_ on BP. **a** STM image of a C_60_ monolayer island on BP obtained with *V*_b_ = 0.80 V and *I* = 30 pA. The image appears like a 2D herringbone stich pattern knitted fabric. C_60_ ball-and-stick models are superimposed on the STM image in the inset to relate the molecular structure of C_60_ with the wave-like contrast. **b** Position-dependent STS spectra obtained on a typical C_60_ molecule within the island. The STS data are recorded at locations around a C_60_ molecule shown in the STM image. The STS d*I* / d*V* spectra in the inset are taken over a larger energy range, to record the band gap of C_60_ molecules. The main unoccupied features due to the LUMO are highlighted in the inset. A low energy shoulder, which has not been observed from previous work, is highlighted by the light-green rectangle. **c** Position-dependent STS spectra obtained for C_60_ molecules approaching an edge of a monolayer island. The colored dots correlate the STS spectra with molecule locations; spectra show that the shoulder feature gradually fades when approaching the island edge (blue and green spectra). **d** Position-dependent STS spectra acquired on the locations across the C_60_/BP edge. The light-green rectangle highlights the energy region where the spectroscopic shoulder feature is observed. (**e**) STS spectroscopic image recorded monitoring the shoulder feature in (**b**) at 0.80 V. The DOS distribution appears delocalized in both the arm-chair and zig-zag directions skirting the indicated C_60_ molecule centers. The black circles in the enlarged image of the insert indicate the four arms connecting C_60_ molecules in the arm-chair direction. The blue circles indicate the two arms in the zig-zag direction. The arms of C_60_ knit the 2D delocalized electronic net of the monolayer. The STM topography images are obtained at 77 K and the d*I*/d*V* spectra and the d*I*/d*V* mapping at 4.5 K

Next, we examine the electronic structure that emerges from the C_60_ monolayer compression. Figure 2a shows an exceptional STM topographic image acquired at a bias of approximately +0.80 V, where the intramolecular contrast of C_60_ vanishes to be replaced by a wave-like supramolecular contrast. The STM image has a herringbone stich knitted fabric pattern with electron density distribution extending deep between the molecules, indicating that the contrast at this energy is dominated by intermolecular electron delocalization. The vanishing of the intramolecular contrast in delocalized 0D, 1D, and 2D structures has been observed for SAMOs of C_60_^22,23^, but such images have not been reported for π-orbitals forming the electronic gap.

We establish the origin of the delocalized electronic structure by recording scanning tunneling spectroscopy (STS) spectra. The inset in Fig. 2b shows a spectrum measured between the valence and conduction bands. It indicates that the C_60_ monolayer is semiconducting with a bandgap of 2.88 eV, very close to that of a C_60_ bilayer or multilayer^38^. The band gap value and the location of the Fermi level within it confirms that the C_60_ monolayer experiences negligible charge transfer upon adsorption on BP. Figure 2b provides STS spectra of the LUMO region, which are measured at four different positions on and around an individual C_60_ molecule selected by considering the symmetry of the adsorption structure (Fig. 2b inset). The spectra are similar at these four sites and show a shoulder peak at the bottom of the LUMO resonance (from 0.67 to 0.82 eV), which distinguishes them from previous measurements. The two sharp peaks, residing at 1.05 and 1.33 eV, respectively, are typical in previously reported LUMO bands for metal supported C_60_ bilayers^38^. The less intense shoulder feature, to which we attribute the herringbone structure of the STM topographic image at 0.8 eV in Fig. 2a, is, however, exceptional to the C_60_ monolayer on BP.

This newly observed shoulder feature in Fig. 2b originates from the electronic hybridization between π-orbitals of C_60_ molecules, as established by a series of spectra measured over single C_60_ molecules in Fig. 2c. The shoulder intensity gradually diminishes as the measuring location approaches the island edge and grows back when the tip returns to the island interior. STS spectra are also acquired on the locations across the C_60_/BP island edge, as shown in Fig. 2d. It is evident that the spectroscopic shoulder feature of C_60_ within the island terminates at the edge C_60_ molecules; after leaving the C_60_ island edge, the spectra show the typical bare BP character^21^. These spectra, thus, compellingly establish that the shoulder belongs to a C_60_ bonding LUMO band, whose density is expected to be maximum within the island bulk and to decrease upon approaching the edge. This conclusion is further confirmed by the spectroscopic d*I*/d*V* map recorded at 0.80 eV (Fig. 2e), where there are appreciable electron densities around the edges of the herringbone topographic contrast. Specifically, the contrast involves four extended arms of C_60_, two to the left and two to the right along the arm-chair direction (indicated by the black circles in the inset of Fig. 2e). These arms link four adjacent C_60_ molecules in the arm-chair direction. In the zig-zag direction, there is also a high intensity contrast (indicated by the blue circles in the inset of Fig. 2e) that links two vertically adjacent C_60_ molecules with one arm on each side. These six arms are responsible for the weak bright contrast observed around single C_60_ molecules mentioned in Fig. 1b. Importantly, the electronic interaction corresponding to the arms form a delocalized 2D electron density network, in sharp contrast to the d*I*/d*V* mapping images of the two commonly observed 1.05 and 1.33 eV LUMO states (Supplementary Fig. 1) where electron densities are localized on single molecules.

### Electronic hybridization among C_60_ on BP surface

We confirm the experimentally observed formation of the delocalized LUMO band by reproducing it with DFT calculations. The calculations are performed for both the C_60_ monolayers with BP substrate and without BP substrate using the supercell shown in Fig. 1f. As shown in Supplementary Fig. 2, the energies and dispersions of the LUMO bands of two C_60_ molecules in the supercell does not appreciably change upon the removal of BP substrate. Furthermore, removing the BP substrate does not cause appreciable change in the partial density of states of C_60_ monolayer (Supplementary Fig. 3). In light of these comparisons, we conclude that DFT results of the isolated C_60_ monolayer without BP substrate fully capture the essential features of the NFE band formation. As shown in Supplementary Fig. 2a, the inclusion of the BP substrate in the electronic structure calculation introduces several substrate bands in the proximity of the C_60_ LUMO bands, which may blur the NFE LUMO band we intended to present. Therefore, we only provide DFT results of the fully relaxed C_60_ monolayer without BP substrate in the following discussions.

**Fig. 3:**
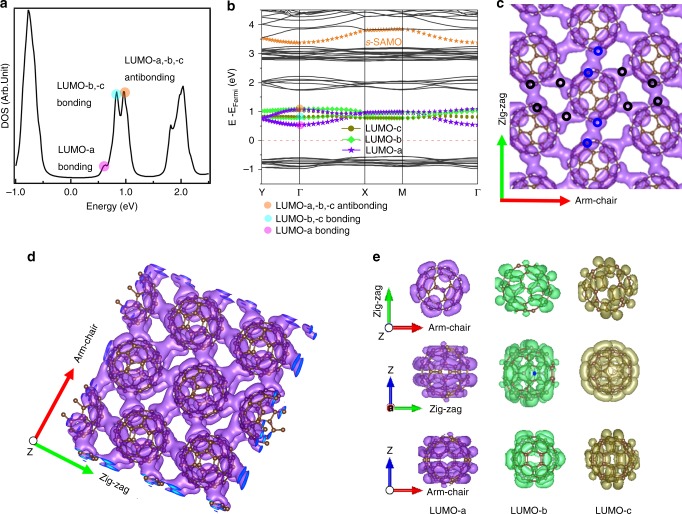
Nearly-free-electron like LUMO band of C_60_ monolayer on BP. **a** DFT calculated DOS of an unsupported C_60_ monolayer lattice with the same geometry as on a BP support, shown in Fig. 1f. The calculated DOS reproduces the two major peaks and one shoulder for the LUMO complex in the STS data. **b** The calculated band structure of the C_60_ lattice in Fig. 1f. The three pairs of bonding–antibonding bands of LUMO-a, -b, and -c are represented by purple, bright green and dark green colors, respectively. The band formed by the superatom *s*-SAMO orbitals, is indicated in orange. Most importantly, the LUMO-a band is as dispersive as the NFE s-SAMO band. **c** The spatial distribution of wave function square of the LUMO-a bonding band at the Γ point. The distribution shows a 2D delocalized character as observed in the STS image (Fig. 2e). The black circles highlight the four arms between adjacent molecules along the arm-chair direction, where the bonding interaction dominates. The blue circles highlight the two arms along zig-zag direction. The iso-surface value of the image is 0.0005 and the largest value is 0.02. **d** 3D image of Fig. 3c shows that the 2D delocalized probability density distribution has two layers in real space. **e** The top and side views of spatial distributions of LUMO-a, -b, -c orbitals of an isolated C_60_ molecule with the same geometry as on BP surface. Z represents the direction vertical to the plane determined by the arm-chair and zig-zag directions. It is evident that the in-plane C atoms in the LUMO-a state have high density in the molecular plane and thus contribute to intermolecular interactions. By contrast The LUMO-b and -c have density that projects above and below the plane, and hence, their intermolecular hybridization is weak

Figure 3a plots the calculated density of states (DOS) of the unsupported C_60_ monolayer. The LUMO region spans an energy range of over 0.5 eV, and consists of a shoulder and two dominant peaks at higher energy; this well reproduces the three features observed in our d*I*/d*V* spectra (Fig. 2b). The band structure plot (Fig. 3b) establishes the band contributions to the DOS peaks. For an isolated C_60_, the LUMO orbitals are triply-degenerate (Supplementary Fig. 4)^39^. LUMO-a, -b, -c derive from the LUMO orbitals of a free molecule when the degeneracy is broken by nonisotropic environment such as caused by the intermolecular and molecule-substrate interactions. The LUMO-a, -b, -c label is normally used to identify the three orbitals that can be identified when the perturbations are sufficiently strong, to distinguish their origin from lowest energy state to the higher ones^19^. Figure 3b is the calculated band structure of C_60_ monolayer with the structures shown in Fig. 1f. Here, the supercell contains two C_60_ molecules and those LUMO orbitals thus form six LUMO bands (three pairs of bonding–antibonding bands) in the monolayer. The dispersion and the energy levels of those bands are determined by C_60_–C_60_ interactions within the monolayer. The lowest unoccupied energy at the Γ point belongs to the LUMO-a bonding band, which results into the shoulder in the DOS plot (marked with the violent circle in Fig. 3a) and corresponds to the shoulder (~0.78 eV) recorded in our d*I*/d*V* STS spectra (Fig. 2b). Those two higher-energy peaks marked with cyan and light orange circles in the DOS plot shown in Fig. 3a are ascribed to the LUMO-b, -c bonding bands and the LUMO-a, -b, -c antibonding bands, respectively, which reproduce the two major d*I*/d*V* peaks at 1.05 and 1.33 eV (Fig. 2b). More details on assignments of the two sharp peaks in the d*I* / d*V* STS spectra are presented in Supplementary Fig. 5.

The compelling result of the band structure calculation is that the LUMO-a bonding band is highly dispersive, characterized with the electron effective masses (m*) of 0.70 *m*_e_ and 0.53 *m*_e_ along the Γ-*X* (zig-zag) and Γ-*Y* (arm-chair) directions, respectively (Table 1). We further confirm the rather small DFT calculated m* in C_60_ monolayer by performing complementary tight binding calculations (Supplementary Note 1 and Supplementary Fig. 6). These calculations give a bandwidth of 0.64 eV for the LUMO-a band and m* values of 0.96 *m*_e_ along the Γ-*X* (zig-zag) and 0.32 *m*_e_ along Γ-*Y* (arm-chair) directions. These results indicate electrons excited or injected into the 2D delocalized LUMO-a band behave like free electrons, which is consistent with the 2D herringbone network appearance of the d*I*/d*V* image (Fig. 2e). A real-space plot of the wave function norm of the 0.57 eV shoulder state (Fig. 3c) reveals wave function overlaps among the four arms of each C_60_ molecule along the arm-chair direction and two arms along the zig-zag direction, accounting for the experimental observation (Fig. 2e). Figure 3d reveals that 2D delocalized charge distribution is a two-layer structure parallel to the surface in real space. The LUMO-b and LUMO-c bands have larger effective masses (Table 1) and their wave functions are more localized to the C_60_ molecules.

**Table 1:** Effective masses and mobilities

	Effective mass, m* (zig-zag)	Effective mass, m* (arm-chair)	Mobility (cm^2^ V^−1^ s^−1^) (zig-zag) (band-like transport model)	Mobility (cm^2^ V^−1^ s^−1^) (arm-chair) (band-like transport model)	Mobility (cm^2^ V^−1^ s^−1^) (arm-chair) (hopping transport model)
LUMO-*a*	0.70	0.53	201–224	339–440	14.7
LUMO-*b*	1.37	3.18	152–177	160–248	8.3
LUMO-*c*	3.16	3.82	135–229	173–281	4.4

The effective masses and mobilities (band-like transport and hopping transport mechanisms) of electron along different transport directions (zig-zag and arm-chair) for the LUMO-a, -b, -c bands

### Origin of the NFE LUMO-a band

A highly delocalized band, such as LUMO-a, is unprecedented for a molecular monolayer bound by vdW forces; it is also significant that it is specific to LUMO-a only. To pinpoint the origin of the NFE bands, in Fig. 3e we first examine the top and side views of probability densities of the LUMO-a, -b, -c orbitals of an isolated C_60_ molecule, with the molecular orientation that is related to experiments. The difference between the LUMO orbitals can be seen by their probability densities with respect to the molecular plane. Specifically, the LUMO-a orbital is primed to hybridize within the molecular plane (Fig. 3e), but the LUMO-b and -c have densities projecting above and below the plane, and hence their intermolecular hybridizations within the molecular plane are weak (Fig. 3e). The calculations show that the unique C_60_ lattice on BP surface favors the in-plane hybridization of probability density of the LUMO-a orbital resulting in the NFE band.

**Fig. 4:**
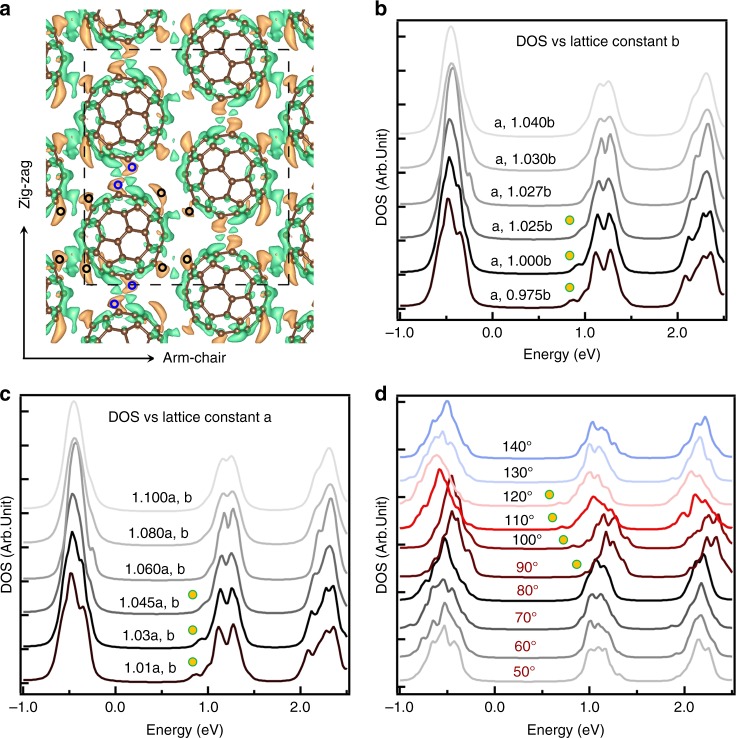
The origin of the nearly-free-electron like LUMO-a band in a C_60_ monolayer. **a** The intermolecular differential probability density [(total − (C_601_ + C_602_ + C_603_ + C_604_); C_601_, C_602_, C_603_, C_604_ represent the four C_60_ molecules in the calculated unit cell indicated by the black dashed rectangle] of a C_60_ monolayer in Fig. 1f. The green and orange iso-surfaces represent the electron donation and accumulation regions, respectively. The results show a probability density sharing redistribution where one arm donates charge and the neighboring molecule arm accepts it. It can be seen that the intermolecular hybridization occurs between molecules that are arranged such that their relative *h*:*h* bond orientations alternate. The iso-surface value of the image is 0.0001 and the maximum value is 0.033. **b**, **c** The appearance of the NFE LUMO-a bonding band shoulder by compression of the lattice constant, *a*, along the zig-zag and *b*, the arm-chair direction; the *h*:*h* bond orientation *φ* is kept constant. The yellow dots with green circle indicate the NFE band density. The trend is that the NFE-shoulder disappears as the lattice constant increases with distortion in *b* being more sensitive than *a*. **d** The appearance of the NFE LUMO-a bonding band shoulder by varying the *h*:*h* relative orientation angle *φ* with the lattice constants *a* and *b* kept constant. The yellow dots indicate the NFE state exists for only a small range of *φ* from 90° to 120° overlapping with the experimentally observed *φ* = 99.2 ± 0.4°. The results show that both the relative orientation of C_60_ molecules and the intermolecular distance play important roles in the formation of the NFE state

The detailed features of the in-plane hybridization can be recognized by plotting the intermolecular DCD of LUMO-a of the monolayer. Figure 4a shows how the delocalized band forms through intermolecular density sharing between C_60_ molecules. In particular, the hybridization occurs at interfaces between molecules along the arm-chair and zig-zag directions (indicated by black and blue circles). This is consistent with the total of six extended arms of C_60_ intermolecular interactions that are observed in d*I*/d*V* mapping image (Fig. 2e). The chirality of the arms along the zig-zag direction is also well reproduced.

### Dependence of the NFE LUMO-a band on C_60_ monolayer topography

The single molecule wave functions and their interactions give clues for the formation of the NFE LUMO-a band. To illustrate the roles of the unique compressive C_60_ molecular lattice that is imposed by the BP, i.e., the intermolecular distance and orientation within the 2D lattice, we calculate the DOS of a C_60_ monolayer, without the substrate, for different lattice constants, *a* and *b*, and mutual orientation angles, *φ*, that are indicated in Fig. 1f. The calculation results are presented in Fig. 4b–d. Figure 4b, c shows that the lattice constant compression along the arm-chair direction, *b*, is critical for enhancing the DOS of the NFE-shoulder state. The NFE-shoulder gradually fades out upon increasing *b* and disappears for 1.03 × *b*, and larger, corresponding to *d*_2_ of ~10.11 Å. The shoulder state is less sensitive upon elongating the lattice constant, *a*, because in the zig-zag direction the lattice is more heavily compressed than the arm-chair direction when the C_60_ monolayer lattice is constructed by the BP template.

Moreover, the calculations show that the lattice compression alone is not sufficient for producing the NFE band; the intermolecular orientation matters. Figure 4d shows the DOS of the monolayer upon varying the intermolecular orientation angle *φ*, while keeping the lattice at a constant *a* and *b*. Varying *φ* has a more complex effect on the distribution of the DOS amplitudes than for varying lattice constants. It is evident, however, that when *φ* is in the range from 90° to 120°, the LUMO peak distribution becomes much broader and small peaks clearly appear in the energy region below the noninteracting LUMO states. The NFE band features are only pronounced within a certain range of angles where the LUMO-a orbitals of adjacent C_60_ molecules have a stronger π-π wave function overlaps (Supplementary Fig. 7). For the C_60_ monolayer on BP, the theoretically and experimentally observed *φ* are 97.3° and 99.2°, respectively. These angles are within the angular window of an appreciable intermolecular interaction, indicating that BP substrate imposes a template that favors inter-C_60_ hybridization. This angle dependence also explains why within C_60_ bulk solids where the intermolecular distance of 10.04 Å is close to the critical distance^34^, the dispersive LUMO bands have not been found. In the case of C_60_ solids, molecules rotate at room temperature, resulting in a random orientation of adjacent molecules that does not support band formation and delocalization. The results explain how the relative C_60_ orientation and compression that are enforced by the BP substrate lead to the intermolecular electronic hybridization of the LUMO-a into the NFE band that is imaged in experiments.

### Carrier mobility

Next, we examine possible novel physical properties that are embodied in the dispersive band of the C_60_ monolayer. The discovery and imaging of the delocalized LUMO-a band suggest that it could enable band-like transport, rather than the well documented hopping mode found in C_60_ aggregates^40^. Table 1 shows the effective masses and calculated electron mobilities using phonon-limited band-like and carrier hopping transport models^40,41^. The actual carrier mobility is difficult to measure by conventional means because the conduction band minimum of BP is below that of C_60_. The theoretical mobilities of LUMO-a are 201 to 224 cm^2^ V^−1^ s^−1^ at 300 K along zig-zag and increase up to 339 to 440 cm^2^V^−1^s^−1^ in the arm-chair direction. These values are comparable to some covalent 2D inorganic solids such as MoS_2_^42^, which is surprisingly high for an organic molecular monolayer that forms through vdW interactions. Carrier mobilities are affected by various processes, thus the predicted values can be different than the measured ones. Nevertheless, it is meaningful to compare theoretical values for a range of molecular layers. Both the calculated hopping (14.7 cm^2^ V^−1^ s^−1^) and the band-like mobility (201–223 cm^2^ V^−1^ s^−1^) of LUMO-a are several times the values calculated for pentacene mono- (hopping, ~10–15 cm^2^ V^−1^ s^−1^) and bi-layers (band-like, 9–65 cm^2^ V^−1^ s^−1^) transport^40^, where the molecular arrangement does not favor effective π-π interaction in the conduction band. The band-like mobilities of LUMO-b and -c also appear to be high. However, the band-like transport mechanism is probably not applicable owing to their modest hybridization within the monolayer. Their hopping mobilities are calculated to be 4.4–8.3 cm^2^ V^−1^ s^−1^, consistent with previous experimental and theoretical values for C_60_ bands with unfavorable intermolecular interactions^9^.

## Discussion

We have provided the experimental evidence and the theoretical understanding for an unusually dispersive NFE-like conduction band of a C_60_ monolayer, which we attribute to a favorable vdW templating of C_60_ molecules that enhance *π*–*π* interactions. In the literature on organic electronics, there are many discussions of π-π interactions, and how they might enhance charge transport, without much actual evidence that NFE band could be induced by π-π interactions. In this work, we show that it does happen for a molecule like C_60_ if the π-π interactions are favored by an appropriate substrate template. The first realization of the NFE conduction band in a C_60_ monolayer presents a new target for exploring the influence of structure on strongly correlated phenomena, *e.g*., insulating, metallic, superconducting and even magnetic phases^5,6,43,44^, in this material. The fact that the π-π vdW interactions could trigger the NFE-like electronic band formation implies that templating of intermolecular interactions by vdW forces on otherwise weakly interacting substrates^45^ provides a promising strategy for tailoring remarkable electronic properties in organic materials for electronics and optoelectronics^2–4,46^.

## Methods

### Sample preparation and STM/STS measurements

The BP crystals are self-grown using a chemical vapor transport (CVT) method [red phosphorus, tin iodide (SnI_4_), and tin powders as the starting materials] in a two-zone tube furnace in a temperature gradient of 600–540 °C. The STM and spectroscopy experiments are carried out in an ultrahigh vacuum low temperature STM system (CreaTec). Prior to STM experiments, the BP crystals are cleaved in-situ in a preparation chamber under ultrahigh vacuum at room temperature (RT). C_60_ molecules (99.9% purity, Aldrich) are sublimated from a resistively heated evaporator onto a freshly prepared BP surface. The room-temperature sample is then immediately transferred into the STM chamber, and cooled down to 77 and/or 4.5 K. The adsorption structure of C_60_ molecules within the monolayer is stable at LN_2_ temperature. STM topographic images are acquired in the constant-current mode. The d*I*/d*V* spectra are measured using the standard lock-in technique with a bias modulation of 15 mV at 321.333 Hz. The STM tips are chemically etched tungsten or mechanically cut Pt-Ir wires, which are further calibrated spectroscopically against the Shockley surface states of cleaned Cu(111) or Au(111) surfaces before being utilized on C_60_/BP.

### DFT calculations

First-principles DFT calculations are performed using the Vienna Ab initio Simulation Package^47,48^. The Perdew-Burke-Ernzerhof (PBE) exchange-correlation functional^49^ along with the projector-augmented wave potentials^50,51^ are used for the self-consistent total energy calculations and geometry optimization. The energy cutoff for the plane-wave basis is set to 400 eV for all calculations. In the band structure calculation, a 7 × 5 × 1 K-mesh is adopted to sample the first BZ of the conventional unit cell of the slab of C_60_; 60 points are collected along each high symmetry line in reciprocal space. vdW interactions are considered at the vdW-DF level when optimizing the system geometry^52,53^. With the optB86b-vdW exchange functional, the optimized lattice constant is in good agreement with the experimental values for BP [3.3 Å (zig-zag) and 4.3 Å (arm-chair)]. The unit cell is optimized fully by letting all atoms in the cell to relax until the residual force per atom is <0.01 eV Å^−1^. A 15 Å vacuum layer separates the neighboring slabs of C_60_ monolayer in vacuum and when deposited on BP. We use a slab model composed of 3 layers of BP to simulate the BP surface.

### Charge carrier mobility calculations

Phonon-limited carrier mobility in C_60_ monolayer with a finite effective thickness *W*_eff_ is expressed as Eq. 1:^41,54–57^

1 $$\mu _{{\mathrm{film}}} = \frac{{{\mathrm{\pi e}}\hbar ^4C_{{\mathrm{film}}}}}{{\sqrt 2 \left( {k_{\rm{B}}T} \right)^{3/2}(m ^\ast )^{5/2}(E_1^i)^2}}F.$$

Here, m^*^ represents the effective mass along the transport direction and *E*_1_ is the deformation potential constant of the VBM (hole) or CBM (electron) along the transport direction; it is determined by $E_1^i = \Delta V_i{{/}}(\Delta {{l/}}l_0)$. Here Δ*V*_*i*_ is the energy change of the *i*th band under proper compressive and tensile strain (by a step $\frac{{\Delta {{l}}}}{{l_0}} = 0{\mathrm{.5\% }}$), *l*_0_ is the corresponding lattice constant along the transport direction, and Δ*l* is the deformation of the lattice constant. The variable *C*_film_ is the elastic modulus of the longitudinal strain in the propagation direction, which is derived by (*E* − *E*_0_)/*V*_0_ = *C*(Δ*l*/*l*_0_)^2^/2; *E* represents the total energy and *V*_0_ represents the lattice volume at the equilibrium for a 2D system. A crossover function *F* bridges the 2D and 3D cases, and is estimated by Eq 2:

2 $$F \equiv \frac{{\mathop {\sum}_n {\left\{ {\frac{{\sqrt \pi }}{2}\left[ {{\mathrm{1 - erf}}\left( {\Omega \left( n \right)} \right)} \right] + \Omega \left( n \right)e^{ - \Omega ^2\left( n \right)}} \right\}} }}{{\mathop {\sum}_n {\left[ 1 + \Omega ^2(n) \right]e^{ - \Omega ^2\left( n \right)}} }},$$

where

3 $$\Omega (n) \equiv \sqrt {\frac{{n^2\pi ^2\hbar ^2}}{{2m ^\ast W_{{\mathrm{eff}}}^2 k_{\mathrm{B}}T}}},$$

The Ω(*n*) (Eq. 3) represents an error function and the summation over integer *n* is due to quantum confinement along the *z*-direction. Effective thickness of the film, *W*_eff_, is expressed by Eq. 4:

4 $$\frac{1}{{W_{{\mathrm{eff}}}}} = {\smallint }_{{\mathrm{ - }}\infty }^{{\mathrm{ + }}\infty } P_i{{(z)}}P_f{(z)\mathrm{d}z} = \mathop {\sum }\limits_n \frac{{\rho _i^n{{(z)}}}}{{{\mathrm{N}}\Delta {{z}}}} \cdot \frac{{\rho _i^n{{(z)}}}}{{{\mathrm{N}}\Delta {{z}}}}\Delta {{z}},$$

Here, *P*(*x*) is the electron probability density along the *z* direction. We divided the space along the *z* direction into *n* parts by Δ*z*. Variable *ρ*^n^(*z*) is the sum of the number of electrons *n*th region along the *z* direction. Here, *N* is the total number of valence electrons in the film, *i* and *f* represent equilibrium and deformed films, respectively. All lattice structural properties and electronic structures in the calculation of carrier mobilities are obtained using the optB86b-vdW functional. The temperature used for the mobility calculations is 300 K.

To evaluate the mobility due to the electron/hole hopping between the adjacent molecules, we apply a treatment expounded by Deng and Goddard^58^, which is based on the Marcus-Hush theory^59,60^. The charge transfer rate, *W*_ij_, between the *i*-th and *j*-th pair of molecules is evaluated as Eq. 5:

5 $$W_{{\mathrm{ij}}} = \frac{{V_{{\mathrm{ij}}}^2}}{\hbar }\left(\frac{\pi }{{\lambda k_{\rm{B}}T}}\right)^{{\mathrm{1/2}}}{\mathrm{exp}}^{ - \frac{\lambda }{{4k_{\rm{B}}T}}},$$

where *V*_ij_ is the coupling matrix element and *λ* is the reorganization energy. By assuming that the charge transfers between pairs of molecules are independent of each other, we can obtain the diffusion constant as Eq. 6:

6 $${\mathrm{D = }}\left\langle {\frac{1}{{2d}}\mathop {\sum }\limits_j r_{{\mathrm{ij}}}^2W_{{\mathrm{ij}}}P_{{\mathrm{ij}}}} \right\rangle _{i = {\mathrm{1,2}}};P_{{\mathrm{ij}}}{\mathrm{ = }}\frac{{W_{{\mathrm{ij}}}}}{{\mathop {\sum }\nolimits^ W_{{\mathrm{ij}}}}},$$

where for 〈…〉_*i* = 1,2_ we take the mean value over two independent molecules in a unit cell, and *j* runs over six neighboring molecules to the i-th molecule. *r*_ij_ is the distance between adjacent molecules and *d* *=* 2 is the system dimension. The Einstein’s relation gives the mobility in the form of Eq. 7:

7 $$\mu \, {\mathrm{ = }}\frac{e}{{k_BT}}{\mathrm{D}}.$$

We take the reorganization energy of single C_60_ molecule to be *λ* = 0.06eV^61,62^.

## Supplementary information

Supplementary Information

Peer Review File

## Data Availability

The data that support the findings of this study are available from the corresponding authors upon reasonable request.
