# Supplementary material for: Realizing nearly-free-electron like conduction band in a molecular film through mediating intermolecular van der Waals interactions
Source: Nat Commun. 2019 Jul 29;10:3374. doi: 10.1038/s41467-019-11300-y (PMC6662711; doi:10.1038/s41467-019-11300-y)
Supplement: Supplementary file 1 — Supplementary Information [file 41467_2019_11300_MOESM1_ESM.pdf]

Supplementary Information for

Realizing nearly-free-electron like conduction band in a molecular film through mediating intermolecular van der Waals interactions

Authors

Xingxia Cui[1†], Ding Han[1†], Hongli Guo[1†], Linwei Zhou[2], Jingsi Qiao[2], Qing Liu[1], Zhihao Cui[1], Yafei Li[1], Chungwei Lin[3], Limin Cao[1], Wei Ji[2]*, Hrvoje Petek[4]*, Min Feng[1,5]*

Affiliations

[1]School of Physics and Technology and Key Laboratory of Artificial Micro- and Nano-Structures of Ministry of Education, Wuhan University, Wuhan 430072, China

[2]Beijing Key Laboratory of Optoelectronic Functional Materials & Micro-Nano Devices, Department of Physics, Renmin University of China, Beijing 100872, China

[3]Mitsubishi Electric Research Laboratories, 201 Broadway, Cambridge, MA 02139, USA

[4]Department of Physics and Astronomy and Pittsburgh Quantum Institute, University of Pittsburgh, Pittsburgh, PA 15260, USA

[5]Institute for Advanced Studies, Wuhan University, Wuhan 430072, China

†These authors contributed equally to this work.
*Corresponding author. Email: wji@ruc.edu.cn (W.J.); petek@pitt.edu (H.P.); fengmin@whu.edu.cn (M.F.)

Supplementary Figure 1. STS d*I*/d*V* IMAGES OF THE C_{60}/BP RECORDED AT DIFFERENT ENERGY VALUES

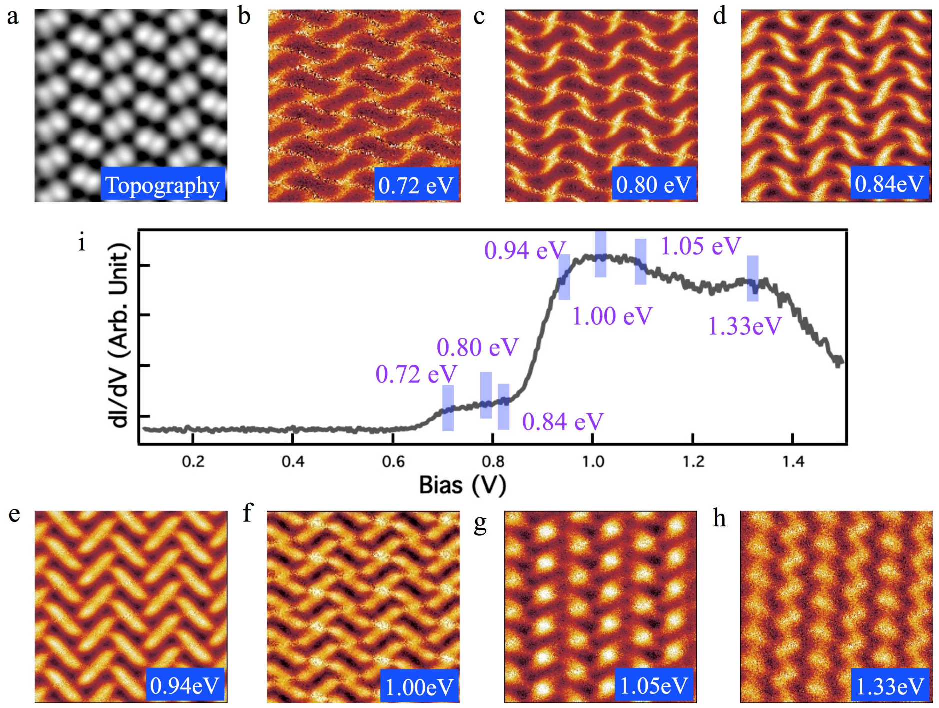

Supplementary Figure 1. STS d*I*/d*V* images of the C_{60}/BP recorded at different energy values in the LUMO energy region. (**a**) to (**h**) STM topographic and STS d*I*/d*V* spectroscopic images of the C_{60} monolayers on BP substrate recorded at the energy values marked in (**i**). (**i**) is the d*I*/d*V* spectrum recorded on a C_{60} molecule within the monolayer. The STS d*I*/d*V* mapping images obtained at the energies of 0.72 eV, 0.80 eV and 0.84 eV, which are within the energy range of the shoulder in (**i**), show that the probability density distribution is delocalized in two-dimensions. The STS images of 1.05 and 1.33 eV, however, show that the probability density distribution at these energies is localized. The images acquired at 0.94 and 1.00 eV represent the DOS distribution that changes from a two-dimensional delocalized character to a localized one.

Supplementary Figure 2. DFT CALCULATED BAND STRUCTURE OF C_{60}/BP AND ISOALTED C_{60} MONOLAYER

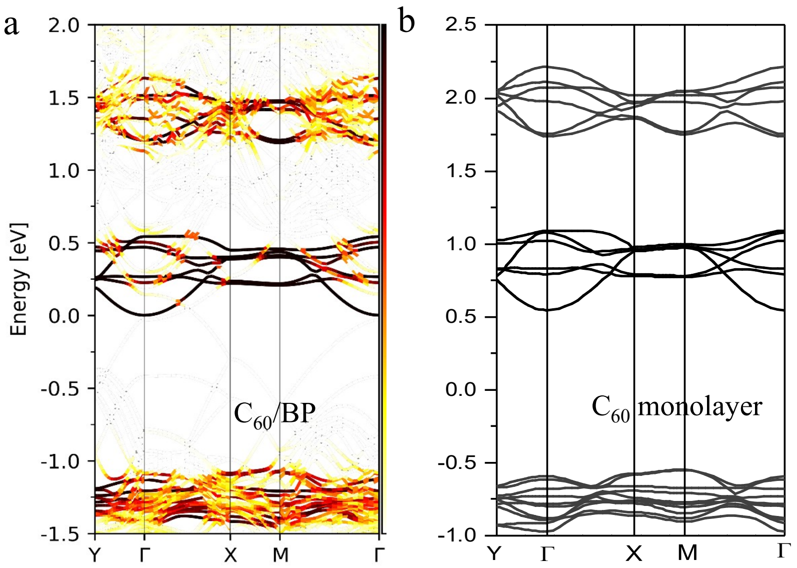

Supplementary Figure 2. DFT calculated C_{60} band structure of C_{60}/BP (a), and isolated C_{60} monolayer (b). In (**a**), the BP bands were projected and removed to show the LUMO bands of C_{60}. The LUMO bands of C_{60} monolayer are essentially the same in both calculations, indicating that BP does not disturb the electronic structures of C_{60} monolayer.

Supplementary Figure 3. DFT CALCULATED DOS OF C_{60}/BP

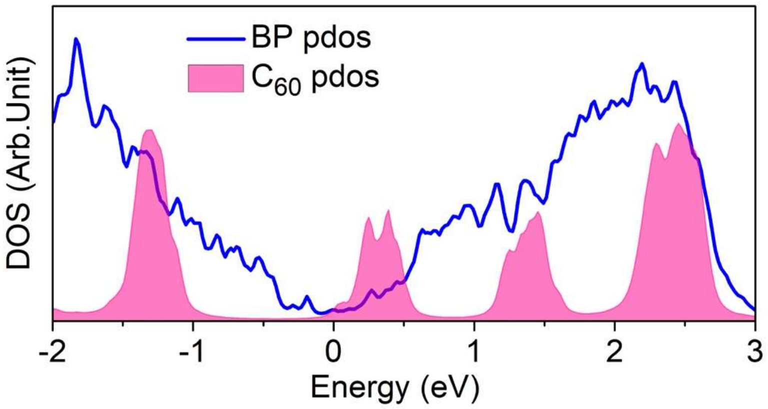

Supplementary Figure 3. DFT calculated DOS of C_{60}/BP. The DFT calculated DOS of C_{60}/BP with the same geometry as that in Fig. 1(**f**). Both the partial density of state (PDOS) of the BP substrate and the C_{60} monolayers are plotted. The PDOS of the BP substrate is similar to that of the bare BP. The PDOS of the C_{60} monolayer is essentially identical to that of the isolated C_{60} lattice (Fig. 3(**a**)). These results show that there is negligible charge transfer between C_{60} and BP substrate.

Supplementary Figure 4. THE ENERGY LEVELS OF LUMO ORBITALS OF AN ISOLATED C_{60} FROM DFT CALCULATIONS

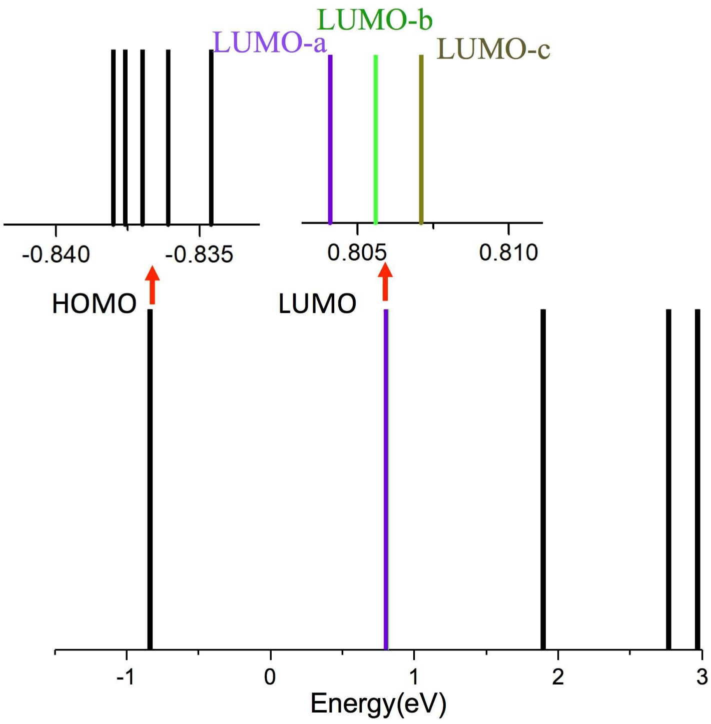

Supplementary Figure 4. The energy levels of LUMO state of an isolated C_{60} from DFT calculations. The LUMO orbitals are composed of three degenerate orbitals with a 3 meV energy difference, which is within the calculation accuracy.

Supplementary Figure 5. DFT CALCULATED BAND STRUCTURE AND THE SPATIAL DISTRIBUTION OF WAVE FUNCTION SQUARE

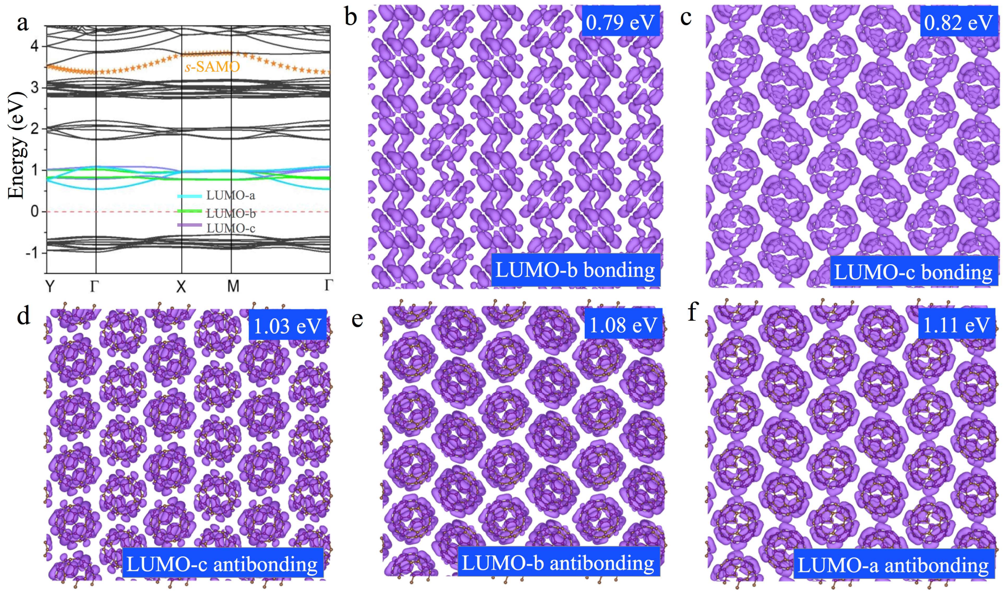

Supplementary Figure 5. DFT calculated spatial distributions of the probability densities of LUMO bands at Γ point. (**a**) DFT calculated band structure of an unsupported C_{60} monolayer with the same geometry as on a BP support, shown in Fig. 1**f**. (**b**) and (**c**) The spatial distributions of probability densities of the LUMO-b, -c bonding bands at Γ point, respectively. (**d**) to (**f**) The spatial distribution of probability densities of the LUMO-a, -b and -c antibonding bands at Γ point. The experimentally measured two major peaks in the STS spectra (Fig. 2**b**), which are located at 1.05 eV and 1.33 eV, originate from the contributions of LUMO-b, -c bonding states and from LUMO-a, -b, -c antibonding states, respectively. The calculated spatial distributions are consistent with the d*I*/d*V* mappings at 1.05 eV and 1.33 eV, shown in Supplementary Fig. 1(g & h).

Supplementary Note 1. TIGHT BINDING CALCULATIONS FOR THE C_{60} MONOLAYER WITH THE EXPERIMENTALLY OBSERVED LATTICE CONSTANTS

Tight binding calculations are performed to double check the consistency of the DFT results. First the transfer integral (t) is calculated within the framework of the Marcus-Hush two-state model,[s1] where the transfer integral for a given electronic level of an isolated neutral molecule is related to its splitting in the dimer. Thus we built a dimer model with the same intermolecular distance and orientation as in the C_{60} film (Fig. 1**f**). This causes LUMO-a to split into a bonding and anti-bonding state, so the transfer integral $t \sim 80$ meV is obtained from Supplementary Equation 1:

$$t = \frac{1}{2}\left(E_{\text{LUMOa-antibonding}} - E_{\text{LUMOa-bonding}}\right) \qquad \text{(Supplementary Equation 1)}$$

We next plot the band structure [Supplementary Fig. 6(b)] of the LUMO-a state based on the experimentally observed lattice constants [Supplementary Fig. 6(a)]. We employ a basic tight-binding Hamiltonian as Supplementary Equation 2:

$$H = \sum_i U_i c_i^{\dagger} c_i + \sum_{ij} t_{ij} c_i^{\dagger} c_j \qquad \text{(Supplementary Equation 2)}$$

where U_i is onsite energy of ith site, and t_{ij} is the transfer integral between sites i and j. For all sites, we used an onsite energy $U = 1.0$ eV and only consider the nearest neighbor hopping with $t = 0.08$ eV. The tight-binding Hamiltonian is expressed as Supplementary Equation 3:

$$h = \begin{pmatrix} 1.0 & f(k) \\ f^{*}(k) & 1.0 \end{pmatrix} \qquad \text{(Supplementary Equation 3)}$$

with $f(k) = -t\sum_{\delta} e^{ik\delta}$, where δ is the vector connecting the site A with the nearest neighbor site B, which are given by $\delta_1 = \frac{a}{2}(1, \sqrt{3})$, $\delta_2 = \frac{a}{2}(1, -\sqrt{3})$, $\delta_3 = \frac{a}{2}(-1, \sqrt{3})$, $\delta_4 = \frac{a}{2}(-1, -\sqrt{3})$. Then we obtain $f(k)$ shown as Supplementary Equation 4:

$$f(k) = -4t\cos\frac{k_x a}{2}\cos\frac{\sqrt{3}k_y a}{2} \qquad \text{(Supplementary Equation 4)}$$

We diagonalize the tight-binding Hamiltonian h to obtain the corresponding eigenvalues shown in Supplementary Equation 5:

$$\varepsilon_{\pm} = 1.0 \pm 4t\cos\frac{k_x a}{2}\cos\frac{\sqrt{3}k_y a}{2} \qquad \text{(Supplementary Equation 5)}$$

From this equation, we see the bandwidth of LUMO-a is $8t$ so that $t = 0.08$ eV gives a 0.64 eV bandwidth. This is consistent with the DFT calculated result of 0.50 eV.

The effective mass of a band is obtained from $\frac{\partial^2 E}{\partial k^2} = \frac{\hbar^2}{m^*}$. If we expand the energy dispersion to the quadratic term, for k_x direction, the quadratic term is $\frac{a^2 k_x^2 t}{2}$, and for k_y direction, it is $\frac{3a^2 k_y^2 t}{2}$. Thus, the effective masses in the x and y directions are $m_x = \frac{\hbar^2}{a^2 t}$ and $m_y = \frac{\hbar^2}{3a^2 t}$. Using $t = 80$ meV and $a = 10$ Å, we obtain $m_x = 0.96\, m_e$, $m_y = 0.32\, m_e$. These calculated effective masses are also consistent with the DFT calculations.

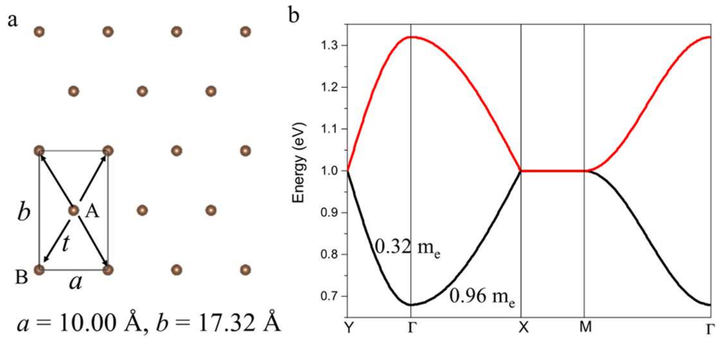

Supplementary Figure 6. Tight binding calculations for the C_{60} monolayer with the experimentally observed lattice constants. (**a**) Lattice constants used in the tight-binding model are a = 10 Å and $b = \sqrt{3}a$ = 17.32 Å. Each C_{60} sits on one site and there are two molecules per supper cell, one molecule sits at the origin (0.0, 0.0) and the other is at (0.5, 0.5). (**b**) The calculated band structure of the LUMO-a orbital using the tight-binding model, which predicts m^* of the LUMO-a bonding band of 0.96 m_e and 0.32 m_e along the Γ-X and Γ-Y directions, respectively.

Supplementary Figure 7. INTERFACIAL PROBABILITY DENSITY DISTRIBUTION BETWEEN ADJACENT C_{60} MOLECULES WITH DIFFERENT φ

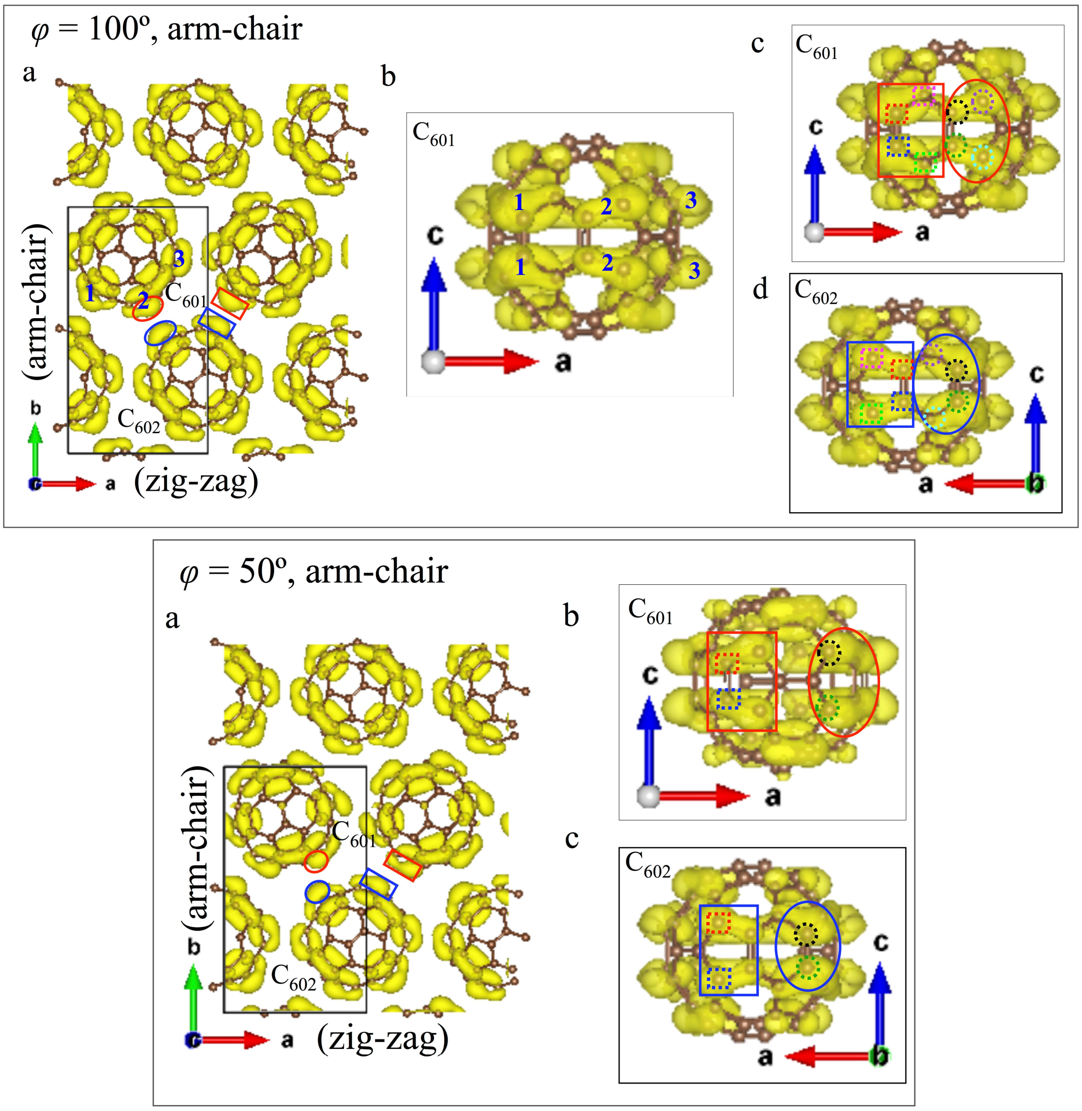

Supplementary Figure 7. Interfacial probability density distribution of adjacent C_{60} molecules with different relative orientation φ. To understand why only in a small range angle φ gives the NFE LUMO-a bands in Fig. 4**d**, the analysis of the probability density distributions at interfaces between molecules, without intermolecular hybridization, is made. Because intermolecular hybridizations alter the interfacial probability density distributions between molecules appreciably, the analysis is challenging. We use the calculated C_{60} lattice to confirm the relative positions of every molecule within the lattice. Then two C_{60} molecules (C_{601} and C_{602}) within one unit cell are isolated to get the partial probability density of LUMO-a for each one. The two partial probability densities are thus added to get the total probability density of the lattice. The top view of the total probability density obtained in this way is represented as image **a** in both top and bottom panels. The side view of each molecule within the unit cell is obtained by utilizing the partial probability density of isolated molecule. With this method we remove the influences of the intermolecular hybridization on the spatial distribution of each molecule and provide a simple way to analyze intermolecular hybridizations.

The top and bottom panels show the analysis for the lattices with the same lattice constants *a*, *b* and with different φ of 100º and 50º. φ of 100º is chosen because it is the closest to the experimentally observed one; φ of 50º is chosen because with this angle, the NFE LUMO-a band is suppressed (Fig. 4**d**). The analysis is made for the intermolecular hybridization along arm-chair direction. The same analysis and conclusion are applied to what happens along the zig-zag direction.

Top panel: (**a**) The top view of the total probability density of the lattice. The ovals and rectangles, with red and blue colors, mark the locations of the probability density at the interfaces between C_{601} and C_{602} along the arm-chair direction. It can be seen that for each molecule, the probability density distribution can be categorized into three types as indicated by number "1", "2" and "3". (**b**) Side view of the probability density distribution of C_{601} (it is the same for C_{602}) highlighting the spatial distribution of "1", "2" and "3". As it has been revealed in Fig. 3**d**, the probability density distribution of LUMO-a has two layers in real space. "1" represents the "large" π probability density shared by three C atoms with a parabolic shape. The parabola opens up in the upper layer and opens down in the lower layer. The "small" π probability density of "2" and "3" has a mutually symmetric shape. Each of them is shared by two C atoms and tilts up in the upper layer and down in the lower layer. As shown in (**b**), the LUMO-a probability distribution, labeled as "1", "2" and "3" follows the spatial orientations of the C-C bonds from which they are originate. At the interface between two C_{60} molecules, the relative orientations of the C-C bonds determine how strong the intermolecular hybridization is. (**c**) and (**d**) Side views, along *b* and *–b* directions respectively, of the interfacial probability density distribution between the C_{601} and C_{602}. The small size dotted rectangles and ovals highlight the C atoms that would share the probability density at the interfaces. As shown in (**c**) and (**d**), all the dotted rectangles and ovals of C_{601} just face the rectangles and ovals of C_{602}, respectively (the correspondence between two C atoms belonging to C_{601} and C_{602} is marked by using the same color), and the related probability density distribution belonging to these C atoms matches well in real space, *i.e.,* the overlap is between aligned bonds This results into strong intermolecular hybridization between two C_{60} molecules this direction.

Bottom panel: All the marks here have the same meanings as in the **Top panel**. (**a**) The top view of the total probability density of the lattice. Compared with (**a**) in the **Top panel** where φ = 100º, it can be immediately seen that the spatial probability density overlap at the interfaces is mismatched in φ = 50º case. (**b**) and (**c**) The side views observing in the *b* and *–b* directions, respectively, of the interfacial probability density distribution between C_{601} and C_{602}. The side views show that the mismatch between the C atoms of C_{601} (dotted rectangles and ovals) prevents interaction with the corresponding C atoms, even though they are in the right position, at C_{602}. This explains the weak intermolecular hybridization when φ=50º and explains the disappearance of the NFE LUMO-a band. The analysis shows that the spatial distribution of the probability density, *i.e.,* their shapes and orientations, makes the intermolecular hybridization favored for certain φ but not for others.

Supplementary references:

1. Deng, W. -Q. & Goddard, W. A. Predictions of hole mobilities in oligoacene organic semiconductors from quantum mechanical calculations. *J. Phys. Chem. B* **108**, 8614-8621, (2004).
